# Unraveling FOXO3a and USP18 Functions in Idiopathic Pulmonary Fibrosis through Single-Cell RNA Sequencing of Mouse and Human Lungs

**DOI:** 10.1055/s-0043-1776697

**Published:** 2023-11-15

**Authors:** Ban Wang, Jichun Pan, Zhonghui Liu

**Affiliations:** 1 Department of Immunology, College of Basic Medical Sciences, Jilin University, Changchun, Peoples' Republic of China; 2 Department of Blood Transfusion, Chinese PLA General Hospital, Beijing, Peoples' Republic of China

**Keywords:** idiopathic pulmonary fibrosis, FOXO3a, USP18, apoptosis, oxidative stress

## Abstract

**Background** Idiopathic pulmonary fibrosis (IPF) is identified as a chronic, progressive lung disease, predominantly marked by enhanced fibroblast proliferation and excessive deposition of extracellular matrix. The intricate interactions between diverse molecular pathways in fibroblasts play a crucial role in driving the pathogenesis of IPF.

**Methods** This research is focused on elucidating the roles of FOXO3a, a transcription factor, and USP18, a ubiquitin-specific protease, in modulating fibroblast functionality in the context of IPF. FOXO3a is well-known for its regulatory effects on cellular responses, including apoptosis and oxidative stress, while USP18 is generally associated with protein deubiquitination.

**Results** Our findings highlight that FOXO3a acts as a critical regulator in controlling fibroblast activation and differentiation, illustrating its vital role in the pathology of IPF. Conversely, USP18 seems to promote fibroblast proliferation and imparts resistance to apoptosis, thereby contributing to the exacerbation of fibrotic processes. The synergistic dysregulation of both FOXO3a and USP18 in fibroblasts was found to significantly contribute to the fibrotic alterations characteristic of IPF.

**Conclusion** Deciphering the complex molecular interactions between FOXO3a and USP18 in fibroblasts provides a deeper understanding of IPF pathogenesis and unveils novel therapeutic avenues, offering a promising potential for not just halting but potentially reversing the progression of this debilitating disease.

## Introduction

Idiopathic pulmonary fibrosis (IPF) is a progressive and often fatal lung disorder characterized by the aberrant proliferation of fibroblasts and consequent deposition of extracellular matrix. 1 This fibrotic process compromises the architecture and function of the lung, leading to decreased oxygen exchange and, ultimately, respiratory failure. The intricacies of its molecular mechanisms, particularly at the cellular level, have remained elusive despite extensive research.

Globally, the incidence of IPF has been observed to increase, affecting predominantly older adults, especially those between 60 and 75 years, with a higher prevalence in men than women. The exact etiology of IPF remains elusive, but genetic predispositions combined with environmental factors, such as smoking or viral infections, have been suggested as potential triggers. At the cellular level, repetitive alveolar epithelial cell injuries, coupled with defective repair mechanisms, lead to the activation of fibroblasts and myofibroblasts that deposit excessive extracellular matrix, resulting in compromised lung function. Current treatment modalities, like antifibrotic agents (e.g., pirfenidone and nintedanib), can slow disease progression but do not offer a cure. Lung transplantation remains the only definitive therapeutic option for end-stage disease, underlining the urgent need for more effective therapeutic strategies.

Single-cell RNA sequencing (scRNA-seq) has emerged as a powerful tool to delineate cellular heterogeneity, offering unprecedented insights into the distinct cellular states and their roles in complex diseases. In the context of lung diseases, scRNA-seq has illuminated the intricate cellular landscape of the lung, allowing researchers to identify previously unknown cell types and states, especially in conditions like asthma, chronic obstructive pulmonary disease, and IPF. By resolving the transcriptomic profiles of individual cells, this technology has facilitated the mapping of cellular transitions during disease progression, uncovering novel pathways and biomarkers. For instance, in IPF, scRNA-seq has been instrumental in highlighting the role of aberrant fibroblast populations in disease pathology. Similarly, in asthma, it has provided insights into the dynamic interactions between airway epithelial cells, immune cells, and other stromal components. As such, scRNA-seq is not only deepening our understanding of lung disease mechanisms but also paving the way for the development of targeted therapies.

The Forkhead Box O (FoxO) subfamily of transcription factors is a group of proteins that play critical roles in a wide variety of cellular processes, including (1) pulmonary homeostasis: FoxOs help to regulate the growth and differentiation of lung cells, as well as the production of mucus and other substances that protect the lungs from infection. (2) Immune and inflammatory responses: FoxOs are involved in the production of proinflammatory cytokines, such as interleukin (IL)-1β and IL-9, which help to fight infection. They also play a role in regulating the innate immune response, which is the body's first line of defense against infection. (3) Various cellular biochemical functions: FoxOs control a variety of cellular biochemical functions, including metabolism, cell cycle progression, and apoptosis (programmed cell death).

In addition to their roles in these important cellular processes, FoxOs are also essential for the development and function of the immune system. They are involved in the maturation and differentiation of B and T lymphocytes, which are the two main types of white blood cells that fight infection. FoxOs are regulated by a variety of factors, including nutrients, growth factors, and stress. When these factors are present, they activate FoxOs, which then bind to DNA and regulate the expression of genes involved in the cellular processes mentioned above. Defects in FoxO signaling have been linked to a variety of diseases, including diabetes, cancer, and chronic lung diseases. Therefore, FoxOs are an important target for research into new therapies for these diseases.

Recently, FOXO3a and USP18, two genes implicated in various cellular processes, have surfaced as potential key regulators in fibroblast functionality linked to IPF. It has been shown that overexpression of FOXO3a reduces TGF-β1-induced fibronectin in lung fibroblast cells without affecting Smad2/3, ISGylation can influence FOXO3a stability, and the USP18/FOXO3a pathway offers a potential treatment for TGF-β1-driven fibrotic diseases. 2 While their generic roles in cellular physiology are recognized, their specific involvement in the context of IPF remains to be elucidated.

Harnessing the abundance of publicly accessible data resources, we embarked on an exhaustive examination of the roles and implications of FOXO3a and USP18 in pulmonary fibrosis. Our investigation encompassed an array of datasets originating from diverse sources: from mouse models to human clinical samples. In the murine datasets, we analyzed transcriptomes from naive mice and contrasted them with those from bleomycin-treated mice, a well-established model mimicking the fibrotic changes seen in IPF. On the human front, our study spanned samples from IPF-afflicted patients as well as lung tissues from healthy controls, enabling a comparative analysis to glean insights into disease-specific alterations. By integrating data across species and health statuses, we aimed to paint a detailed landscape of how FOXO3a and USP18 participate in the intricate molecular dance that culminates in fibrotic transformations characteristic of IPF. This holistic approach is designed not only to shed light on their individual contributions but also to discern any synergistic or antagonistic interactions they might have within the broader context of IPF pathogenesis.

## Results

### Differential Expression of FOXO3 and USP18 in Human Idiopathic Pulmonary Fibrosis Lungs

**Fig. 1:**
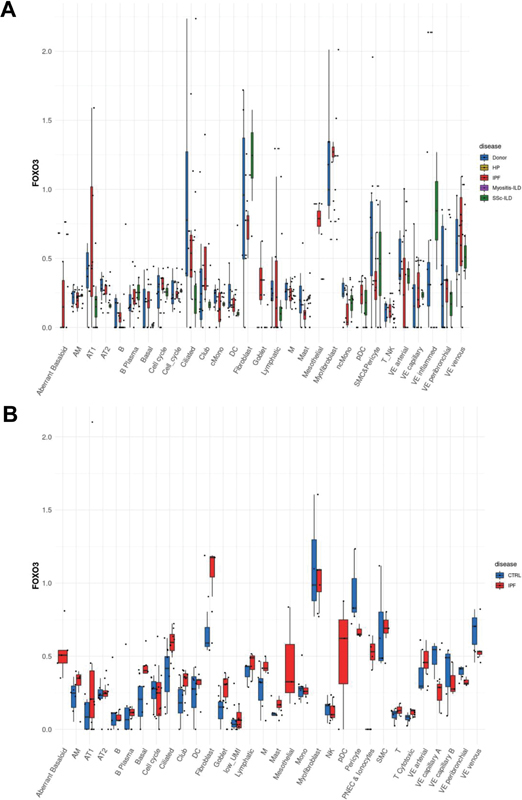
Differential expression of FOXO3 in human IPF lungs across studies. Utilizing single-cell analysis of human lung tissues, this figure depicts the variable expression levels of FOXO3 across different identified cell types from two distinct datasets. The divergent findings from ( **A** ) the Misharin group and ( **B** ) the Lafyatis group are illustrated, highlighting the heterogeneity and complexity of FOXO3 expression in IPF lungs.

**Fig. 2:**
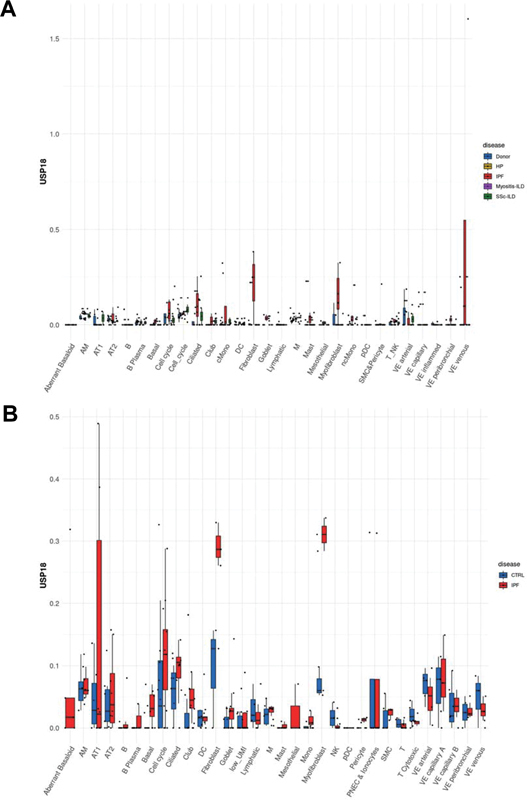
Variation of USP18 expression in human IPF lungs across investigations. Through meticulous single-cell analysis, we demonstrate the distinct expression profiles of USP18 in different cell types present in human lung tissues. Two prominent datasets are compared: ( **A** ) The Misharin group's findings and ( **B** ) those of the Lafyatis group, underscoring the importance of understanding variances in USP18 levels in IPF pathology.

Diving into the nuanced expression patterns of FOXO3 and USP18 in human lung tissues affected by IPF and its related pathologies, we turned to the comprehensive LungMAP public database, an invaluable resource for lung-centric transcriptomics. Initial explorations into this dataset unveiled intriguing discrepancies. Specifically, the Misharin group 3 ( Fig. 1A, B ) documented a noticeable decline in FOXO3 expression in tandem with a surge in USP18 levels within their curated IPF dataset ( Fig. 1A, B ). This stood in stark contrast to the Lafyatis group's data, where FOXO3 showcased an upward trajectory in expression, although the USP18 trends echoed those seen in the Misharin group's findings ( Fig. 2A, B ). 4 The complexity deepened further when examining data from two other prominent research teams that had contributed to the database. Both the Kaminski team and the collaboration between Banovich and Kropski, 5 6 added their own single-cell RNAseq data to this ever-expanding resource. Yet, their reported expression dynamics for FOXO3 and USP18 veered away from the patterns observed in the Misharin and Lafyatis datasets ( Supplementary Figs. S1 and S2 , available in the online version). Such discrepancies highlight the inherent challenges and intricacies in deciphering gene expression patterns across different research platforms, emphasizing the importance of thorough analyses and cross-referencing in understanding IPF's molecular landscape.

### Expression Profiling in Naive Mouse Lungs

**Fig. 3:**
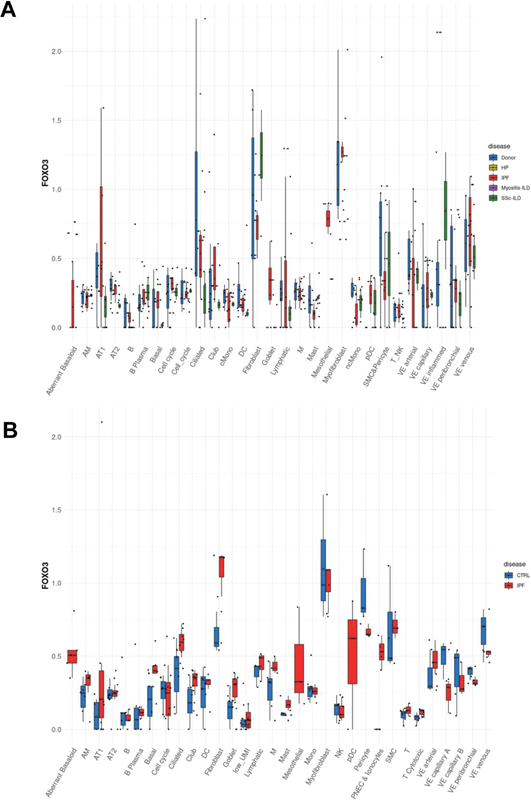
Comprehensive analysis of FOXO3 and USP18 expression in naive mouse lungs. The Tabula Muris dataset offers a comprehensive view of single-cell transcriptomic data from the *Mus musculus* , incorporating nearly 100,000 cells from a diverse range of organs and tissues. This figure contrasts the expression profiles of FOXO3 using two technical methodologies: ( **A** ) microfluidic droplet-based 3′-end counting, which offers a broad survey of cells at a reduced coverage and ( **B** ) FACS-based full-length transcript analysis, which affords increased sensitivity and coverage.

To better comprehend the intricate complexities of IPF and circumnavigate the potential technical inconsistencies inherent to single-cell RNAseq sample preparations, we purposefully directed our focus to the more consistent and replicable environment presented by mouse models. Leveraging the expansive Tabula Muris dataset, 7 a noticeable predilection for Foxo3 expression in stromal cells became apparent ( Fig. 3A, B ). A salient feature that stood out in our findings was the remarkable steadfastness of this expression profile, a trait that persisted irrespective of the choice of single-cell analysis techniques employed. This unwavering consistency was observed not only when leveraging the well-established fluorescence-activated cell sorting (FACS) method, which isolates and categorizes individual cells based on their fluorescent signatures, but also when deploying the advanced drop-seq-based single-cell capture technique. This latter approach is renowned for its precision in concurrently segregating and sequencing individual cells, thereby providing a high-resolution snapshot of cellular heterogeneity. The fact that such diverse methodologies converged on analogous results underscores the inherent robustness of the observed expression patterns and minimizes the likelihood of these observations being mere artifacts of the chosen analysis tool. We also looked at Usp18 expression and came to the same conclusion ( Supplementary Fig. S3 , available in the online version).

### FOXO3 and USP18 Dynamics in Bleomycin-Induced Mouse Lung Fibrosis

**Fig. 4:**
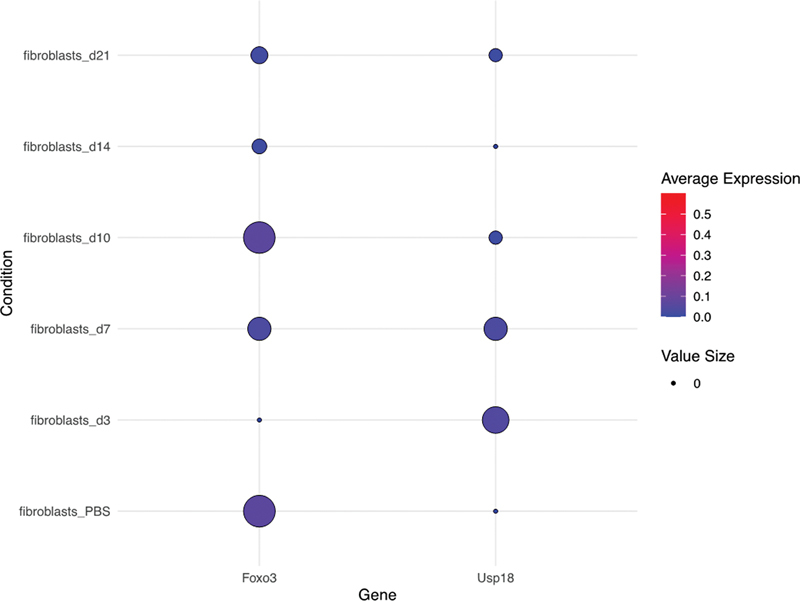
Dynamic Shifts in FOXO3 and USP18 expression following bleomycin treatment in mouse lungs. Presented via dot plots, the gene expression patterns of FOXO3 and USP18 in bleomycin-exposed mouse lungs are showcased. The size of each dot represents the percentage of cells expressing the gene within the fibroblast population, while the dot's color intensity reflects the average gene expression level across all fibroblast cells.

Having confirmed the baseline expression dynamics of Foxo3 and Usp18 in unperturbed murine lung tissue, our investigative journey led us to delve into their potential roles within a mouse paradigm that closely mimics the pathological trajectory of IPF. The widely recognized and frequently utilized bleomycin-induced lung damage model, which subsequently culminates in fibrotic alterations, was our model of choice. An exhaustive combing through a contemporaneous study's single-cell RNAseq dataset unveiled an intriguingly ascending trajectory in Foxo3 expression over a delineated temporal span. 8 Captivatingly, this elevation seemed to exhibit an inverse correlation with the oscillating expression patterns of Usp18 ( Fig. 4 ). Such a contrasting dynamic underscored our burgeoning belief that these genes, in tandem, could be wielding considerable influence over the molecular progression of IPF, especially in the bleomycin-induced lung fibrosis setting in mice.

### Probing Fibroblast Interactions with Varied Cell Types

**Fig. 5:**
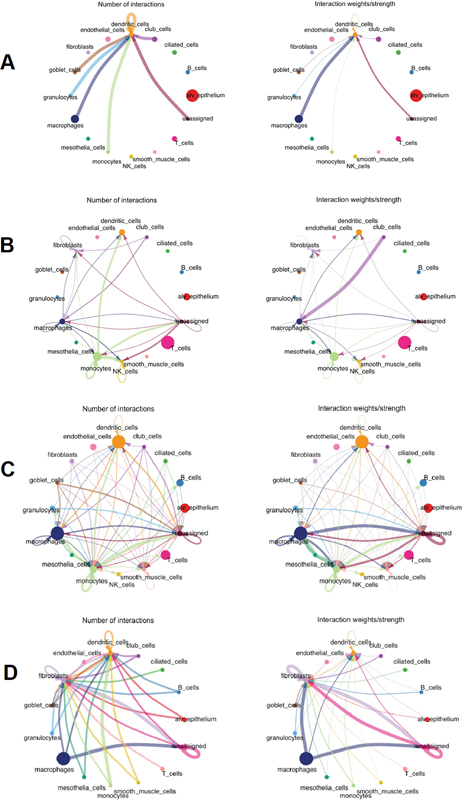
In-depth cell–cell interaction analyses involving fibroblasts and other cellular types. Using the sophisticated CellChat tool, we quantitatively infer and dissect the intercellular communication networks, with a focus on interactions between fibroblasts and other cellular components. This analytical perspective provides vital insights into the cellular dialogue occurring within the tissue landscape.

One of the pivotal strengths of employing single-cell RNAseq data for cellular interaction studies is its unparalleled capability to both reaffirm extant knowledge and simultaneously unveil novel interaction paradigms. Leveraging advanced analytical tools like the CellChat 9 R toolkit, we meticulously parsed through the bleomycin-induced dataset. Our rigorous analysis delineated a conspicuous lack of fibroblast communication with other cellular counterparts under baseline (phosphate-buffered saline) circumstances ( Fig. 5A ). As the fibrotic scenario began to evolve, there was a marked increase in the interactive behavior of fibroblasts, most notably with club cells and monocytes. This heightened cellular interplay wasn't uniform across the course of the disease progression. Instead, it reached peak intensities at distinct intervals following bleomycin induction, specifically on days 3, 7, and 10, as depicted in Fig. 5B, C, D . These staggered, yet pronounced, interaction bursts are more than just random events in the disease timeline. They suggest a temporally coordinated cellular response, with fibroblasts potentially at the helm of this orchestrated dance. The nuanced shifts in fibroblast communication with specific cell types could be pivotal, acting as both markers and influencers of disease stages. Understanding these intricate interaction dynamics offers not only a deeper glimpse into the mechanistic underpinnings of pulmonary fibrosis but also potentially unveils targets for therapeutic interventions, emphasizing the central and influential role fibroblasts assume in guiding the sequence of molecular and cellular events culminating in fibrotic alterations within the lung.

### Corroborating the Involvement of Foxo3 and Usp18 through an External Dataset

**Fig. 6:**
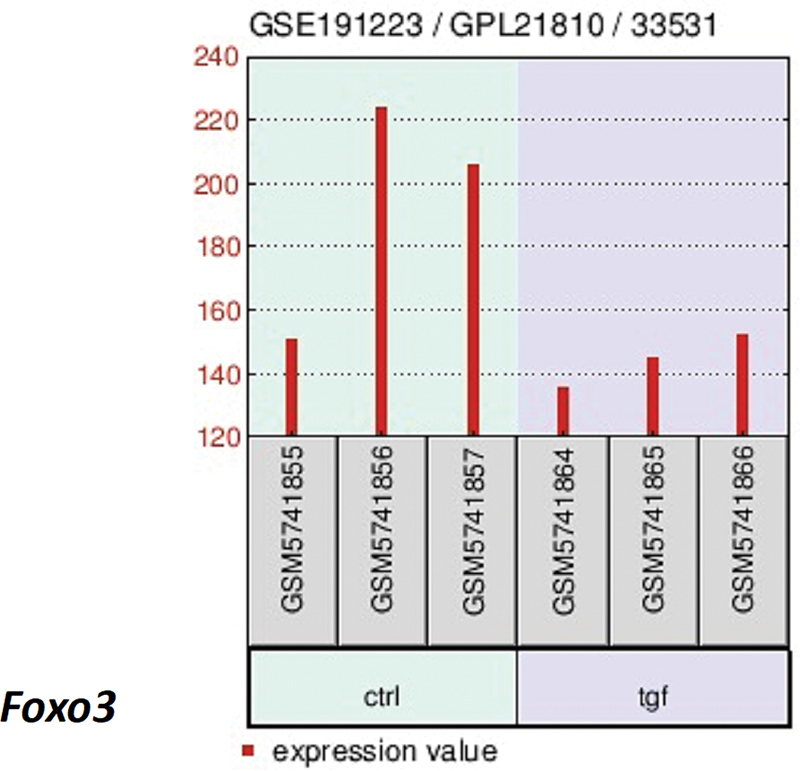
Independent validation of Foxo3′s role in fibrogenesis through microarray analysis. A deep dive into microarray data reveals the modulation of Foxo3 by TGFb, a pivotal mediator in fibrogenesis. This analysis further consolidates the pathogenic role of Foxo3 in pulmonary fibrosis and its potential modulation by fibrogenic factors.

To bolster our understanding of Foxo3 and Usp18 in the context of pulmonary fibrosis, we scoured the GEO database. A pertinent microarray study emerged, focusing on pivotal fibrotic triggers like transforming growth factor-β in fibroblasts. 10 In this investigation, the authors delved into the effects of various cytokine stimulations on fibroblast cellular dynamics, utilizing the NIH 3T3 cell line as a model system. The NIH 3T3, a well-characterized and widely used fibroblast cell line, offers a reliable platform to dissect the cellular responses to different cytokine challenges. A range of treatment conditions were scrutinized, with TGF-β standing out as a particularly significant stimulant. Given TGF-β's established role in fibrosis and its profound influence on fibroblast function and differentiation, its inclusion in our experimental matrix was paramount. By comparing the effects of TGF-β with other cytokine treatments, this study aims to unravel the intricate signaling pathways and downstream cellular responses associated with fibroblast activation and potential transdifferentiation. Upon delving deeper into the dataset, we observed a marked suppression of Foxo3 expression when the cells were treated with TGF-β, as depicted in Fig. 6 . TGF-β, a pivotal molecule in fibrotic processes, seems to have a profound inhibitory effect on Foxo3 expression. This reduction in Foxo3 levels, when juxtaposed with the known functions of the gene, suggests a critical connection. Furthermore, to validate these findings, we did an independent investigation using real-time reverse transcription polymerase chain reaction and observed the same effect of TGF-β ( Supplementary Fig. S4 , available in the online version). Considering Foxo3's roles in various cellular processes, its diminished expression in response to TGF-β raises pertinent questions about its protective or modulatory functions in the fibrotic pathway. Thus, the suppressed Foxo3 expression in the presence of TGF-β may hint at an environment conducive to the progression or exacerbation of lung fibrosis, underscoring the need to further explore the molecular mechanisms and potential therapeutic implications of this interaction.

## Discussion

The expression dynamics of FOXO3 and USP18 in human pulmonary tissues, particularly when influenced by IPF, have been subjects of considerable research intrigue given their postulated impact on disease pathogenesis. Our thorough dive into the LungMAP public database furnished striking variances in the expression profiles of these pivotal genes among IPF-affected individuals. A salient observation from our exploration was the evident dip in FOXO3 levels coinciding with a surge in USP18 in an IPF dataset curated by the Misharin group. However, these findings weren't mirrored across all research spectrums, as evidenced by the contrasting FOXO3 expression patterns presented by the Lafyatis group, even though USP18 trends remained somewhat congruent. Such inconsistent findings spotlight the intricacies woven into IPF's progression, potentially stemming from diverse genetic predispositions, environmental interferences, or the inherent multifaceted nature of the ailment. It is of note that different protocols, digestion enzymes, and buffers can have an impact on the enrichment of certain cell types or even alter gene expression. This is because the choice of these factors can affect the viability and functionality of the cells, as well as the specific molecules that are released from them. For example, the use of different digestion enzymes can result in the selective degradation of certain cell types or molecules. Similarly, the use of different buffers can affect the pH of the environment, which can also influence cell viability and gene expression. It is important to carefully consider the choice of protocols, digestion enzymes, and buffers when performing experiments that involve the enrichment of cell types or the analysis of gene expression. By doing so, researchers can minimize the risk of introducing bias into their results.

Extending our investigative lens to encompass murine models provided an enriched perspective into this enigmatic landscape. The controlled confines of mouse models offer an environment where many of the variables present in human studies—like diverse treatment histories, age-related influences, or concurrent health issues—are essentially absent. Our meticulous analysis of the Tabula Muris dataset unraveled pronounced Foxo3 expression in stromal cells, an observation consistently maintained across disparate single-cell analytical techniques. Such unwavering consistency accentuates the role of cellular biology in dictating gene expression, rather than external technological factors. The implications of such homogeneous findings are profound, laying a robust foundation for ensuing experimental explorations.

The Tabula Muris dataset utilized two different platforms: FACS and droplet-based. FACS is a technique that uses a laser to identify and sort individual cells based on their fluorescent properties. This technique is often used to isolate specific cell types or to enrich cells that express a particular gene or protein. Droplet-based analysis is a newer technique that uses microfluidics to encapsulate individual cells in droplets. Each droplet contains a single cell, and the droplets are then analyzed for their fluorescent properties. This technique is able to analyze a much larger number of cells than FACS, and it is also less disruptive to the cells. The similarity between FACS and droplet-based analysis is that they both allow for the analysis of individual cells. However, there are also some important differences between the two techniques. FACS is a more destructive technique than droplet-based analysis. When cells are sorted by FACS, they are exposed to a laser, which can damage the cells. Droplet-based analysis does not require the use of a laser, so it is less disruptive to the cells. FACS is a more selective technique than droplet-based analysis. FACS can be used to isolate specific cell types or to enrich cells that express a particular gene or protein. Droplet-based analysis is not as selective, but it can be used to analyze a much larger number of cells. We observed consistent gene expression patterns between the two platforms suggesting that the expression profile is not due to any artifacts introduced by the analysis technique.

A testament to this assertion is mirrored in our insights derived from the bleomycin-induced lung injury mouse model, an established standard for mimicking IPF. As we navigated this terrain, the oscillating dynamics between Foxo3 and Usp18 became apparent. The observed uptick in Foxo3 expression, juxtaposed against a diminishing Usp18 trend as the fibrotic process advanced, casts a spotlight on their potential intertwined roles in the fibrotic narrative. The underpinning mechanisms guiding this association, be it a regulatory hierarchy or a synergistic collaboration, offer tantalizing prospects for further scrutiny. Augmenting this narrative is our cell–cell interaction analysis, which unequivocally emphasizes the linchpin role fibroblasts assume in the complex tapestry of pulmonary fibrosis. Their intensified dialogues with club cells and monocytes postinjury might serve as the initial sparks, igniting a destructive cascade with profound repercussions on pulmonary architecture and functionality.

It is of note that our investigation into an independent dataset further bolsters the potential involvement of Foxo3 in pulmonary fibrosis. The downregulation of Foxo3 in the presence of fibrotic initiators such as TGF-b might imply a protective role for Foxo3 against fibrosis, where its absence or reduction paves the way for pathogenesis. As our understanding of these molecular players grows, they not only shed light on IPF's intricate biology but also point toward potential therapeutic targets in its management. A recent study underscores FoxO3′s pivotal role in lung fibrosis and hints at novel therapeutic avenues via FoxO3 manipulation. 11 The authors investigated ex-vivo-cultured fibroblasts from both human IPF lungs and bleomycin-treated mice. This research highlighted a reduced expression of the FoxO3 transcription factor, increased phosphorylation, and its exclusion from the nucleus compared with controls. When standard human lung fibroblasts were exposed to various profibrotic growth factors, similar effects on FoxO3 were observed. Notably, FoxO3 knockdown in these fibroblasts mimicked the phenotype linked with transdifferentiation and heightened proliferation. Mice lacking FoxO3 showed a heightened susceptibility to bleomycin, emphasizing exacerbated fibrosis, declined lung function, and increased mortality. Importantly, activating FoxO3 using UCN-01 reversed the IPF myofibroblast phenotype in vitro and mitigated bleomycin-induced lung fibrosis in vivo.

Following the recent findings on FOXO3a and USP18, their prominence in the context of IPF has become evident. The ability of FOXO3a overexpression to mitigate the effects of TGF-β1, a cytokine central to fibrosis, indicates its potential therapeutic value. Additionally, the modulation of FOXO3a stability through ISGylation hints at avenues where interventions can be applied to stabilize or enhance FOXO3a activity in fibroblasts, potentially halting or reversing fibrotic processes. The USP18/FOXO3a pathway, in particular, stands out as a promising target, especially given the interconnected regulatory mechanisms these two molecules share. From a therapeutic perspective, small molecules or biologics that can modulate the activity of FOXO3a or USP18 can be developed. Such therapeutic agents could either enhance FOXO3a's protective effects against fibronectin accumulation or inhibit deleterious USP18 activities that might exacerbate fibrosis. Additionally, given the potential regulatory interactions between FOXO3a and USP18, combination therapies targeting both molecules simultaneously might offer synergistic benefits. Furthermore, gene therapy approaches, harnessing CRISPR/Cas systems or siRNA methodologies, could be employed to enhance or suppress the expression levels of these genes in the lung fibroblast population. Nonetheless, it is essential to consider the systemic effects of targeting these genes. Given their involvement in multiple cellular processes beyond fibrosis, a targeted delivery system, such as nanoparticle-based drug delivery, might be necessary to ensure the therapeutic agents' specificity to lung fibroblasts and minimize off-target effects. As the field continues to unravel the roles of FOXO3a and USP18 in IPF, it is imperative to move forward with in vivo studies and clinical trials to evaluate the safety, efficacy, and feasibility of these potential therapeutic strategies.

## Methods

### Single-Cell RNAseq Analysis

Single-cell RNA sequencing data were processed and analyzed using the Seurat package 12 4.0 with R (version 4.1.1). The Seurat workflow incorporated multiple stages, including quality control, data filtering, normalization, and identification of highly variable features. Principal Component Analysis was performed to reduce the dimensionality of the data, facilitating accurate clustering of cells into distinct populations. Marker genes for each identified cluster were determined using Seurat's built-in functions. For visualization purposes, Uniform Manifold Approximation and Projection and t-distributed stochastic neighbor embedding plots were generated. Additionally, certain data visualization tasks, especially those requiring specific annotations, were accomplished utilizing resources from both LungMAP and Tabula Muris.

### Cell–Cell Interaction Analysis

To shed light on the complex web of intercellular communication within the analyzed samples, we employed the CellChat9 framework. This tool enabled a comprehensive exploration of the scRNA-seq data, allowing us to pinpoint potential ligand–receptor pairs and, by extension, the signaling pathways they might regulate. Such analyses are vital to infer the potential influence one cell type might exert over another, shaping tissue behavior and disease progression.

### Microarray Data Processing

Raw microarray datasets pertaining to our research were sourced from the Gene Expression Omnibus (GEO) database. Upon retrieval, the data underwent a standardized preprocessing workflow. This included background correction, normalization, and summarization of probe-level intensities to generate gene expression matrices. The subsequent statistical analysis, aimed at identifying differentially expressed genes under various conditions, was facilitated using the GEO2R tool provided by the GEO platform. This allowed for a comparison of expression profiles under distinct experimental conditions, thereby revealing potential genes of interest and their associated regulatory pathways.
