# Supplementary material for: Unraveling FOXO3a and USP18 Functions in Idiopathic Pulmonary Fibrosis through Single-Cell RNA Sequencing of Mouse and Human Lungs
Source: Glob Med Genet. 2023 Nov 15;10(4):301–10. doi: 10.1055/s-0043-1776697 (PMC10651367; doi:10.1055/s-0043-1776697)
Supplement: Supplementary file 1 — Supplementary Material [file 10-1055-s-0043-1776697-s2300060.pdf]

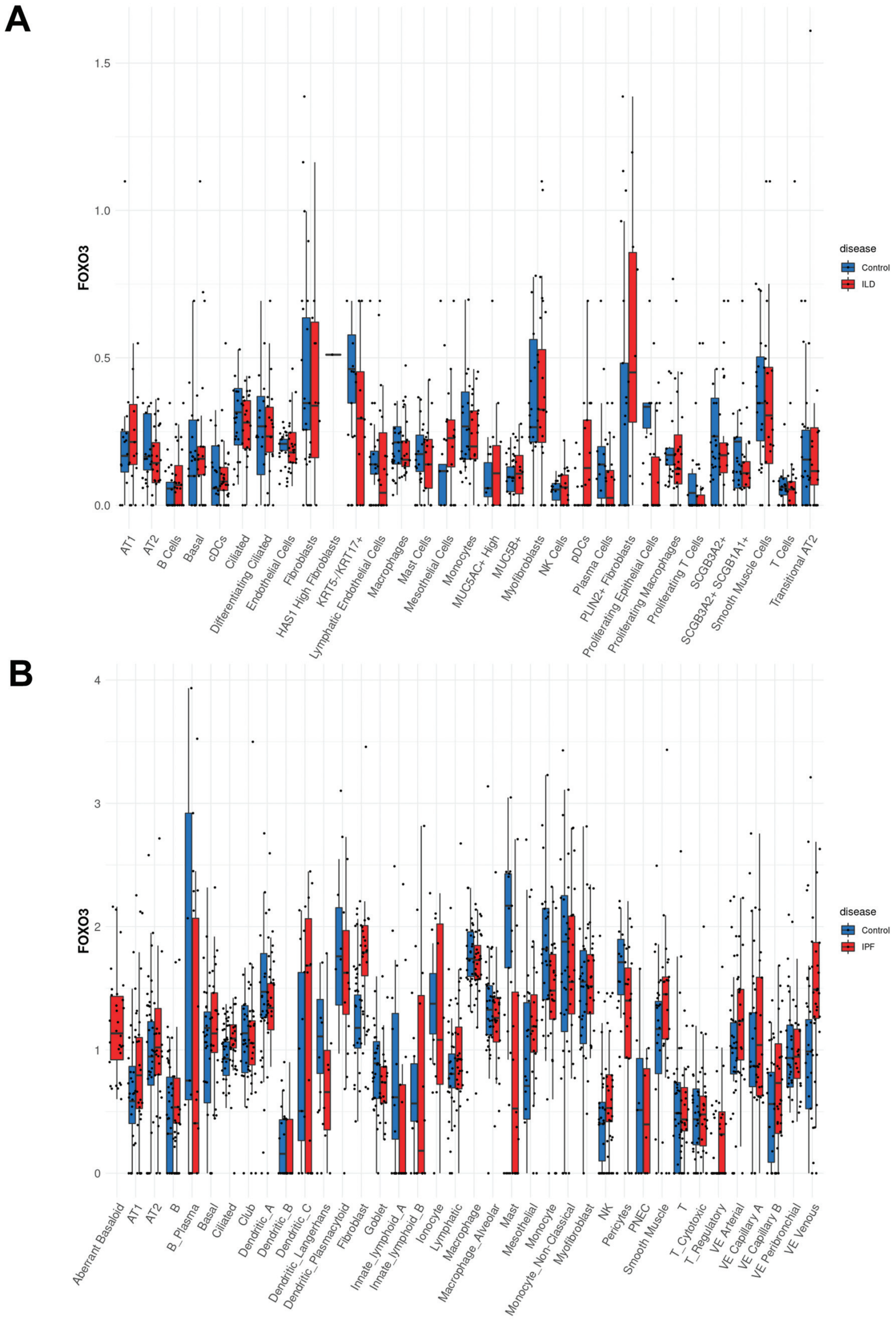

Supplementary Fig. S1 Differential expression of FOXO3 in human IPF lungs across studies.

 © 2023. The Author(s).

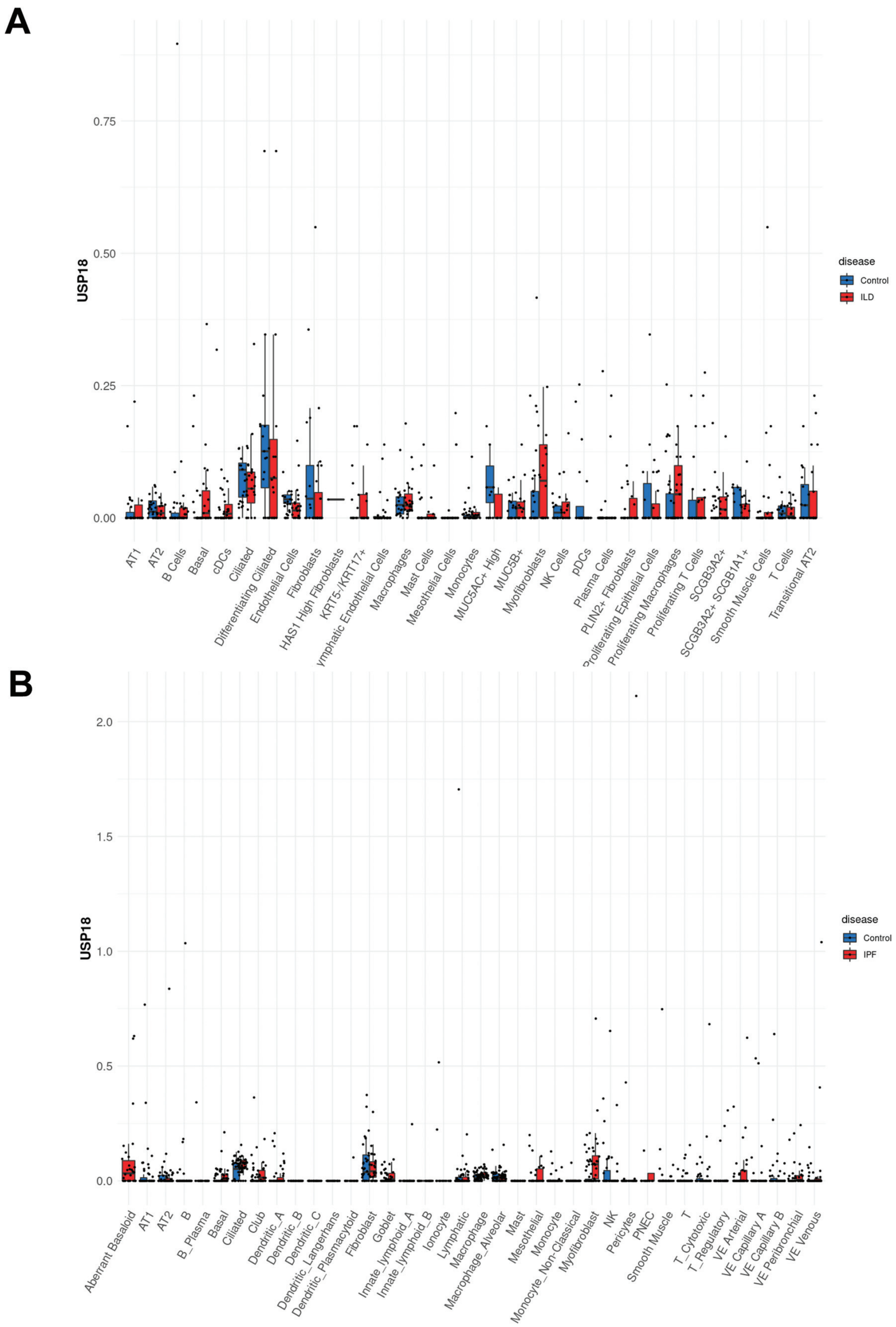

Supplementary Fig. S2 Variation of USP18 expression in human IPF lungs across investigations.

 © 2023. The Author(s).

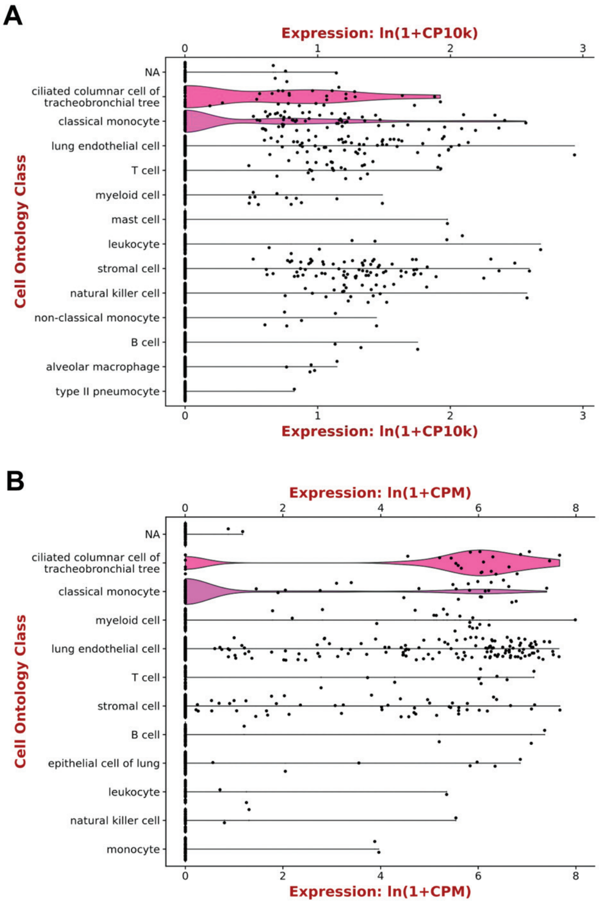

Supplementary Fig. S3 Comprehensive analysis of USP18 expression in naive mouse lungs.

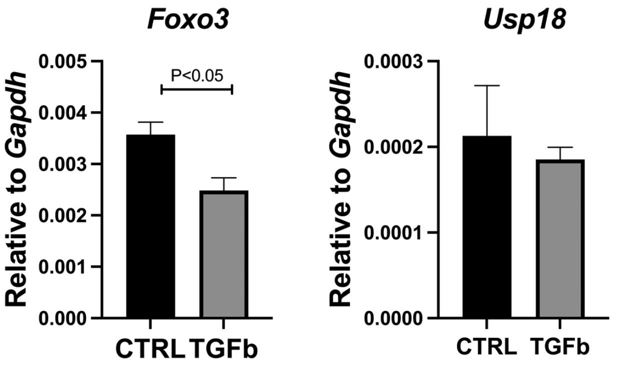

Supplementary Fig. S4 Validation of the TGF-β induced suppression of Foxo3 expression.
